# Quenching the thirst internally: separating miRNA from target mRNA by condensation of AGO1

**DOI:** 10.1093/plcell/koag175

**Published:** 2026-06-11

**Authors:** Pei Qin Ng

**Affiliations:** Assistant Features Editor, The Plant Cell, American Society of Plant Biologists; Department of Plant Sciences, University of Cambridge, Cambridge CB2 3EA, United Kingdom

Water is essential for the survival of terrestrial plants, and dehydration triggers stress responses in them. Prolonged dehydration, such as drought, can be detrimental to growth and survival. Adaptive strategies, such as physiological, metabolic, and gene expression changes are often deployed by plants to cope with dehydration (Blum and Tuberosa 2018).

MicroRNAs (miRNAs) regulate gene expression during stress by binding to their target messenger RNAs (mRNAs), a mechanism known as miRNA-mediated gene silencing. Mature miRNAs are loaded into Argonaute 1 (AGO1), which forms part of the RNA-induced silencing complex (RISC), allowing translation inhibition or cleavage of target transcripts (Vaucheret et al. 2004; Islam et al. 2022). Most studies of dehydration-stress regulation have focused on the miRNAs but not much has been known about the AGO1 regulation under such conditions. Blagojevic et al. (2024) first reported that AGO1 proteins can transition from a freely diffusing state into cytosolic condensates that co-localize with stress granules under heat stress through a process driven by liquid-liquid phase separation (LLPS) a phenomenon similar to oil droplets forming on water. However, the mechanistic insights into the AGO1 condensate formation during dehydration in plants and the impact on dehydration-related miRNA–mRNA interaction have yet to be explored.

In a recent study in *The Plant Cell*, **Hyun Ju Jung and colleagues (2026)** explored the cause of AGO1 condensate formation and how this phenomenon impacts miRNA–mRNA interaction and regulation in the model plant system *Arabidopsis thaliana*. They first investigated the levels of AGO1 protein under dehydration stress conditions, and they observed a 4-fold increase of AGO1 proteins, despite a lower level of *AGO1* mRNA accumulation. To understand the global changes of miRNA and mRNA during dehydration, the authors performed RNA-sequencing and differential expression analysis. For the miRNAs, the authors observed only a mild global change of miRNA levels, with 37 miRNAs downregulated and 48 miRNAs upregulated in both 12- and 24-h dehydration-treated seedlings. As for the mRNA level, only 393 putative target genes were differentially expressed with a ratio change of at least ± 0.2. Collectively, these observations showed that AGO1 abundance does not lead to drastic changes of miRNA and mRNA levels globally, raising the question of how the inverse correlation between miRNA and target mRNA level is maintained.

To test for the deviation from the inverse correlation of miRNA and target mRNA levels, Jung et al. (2026) performed global correlation analysis of the miRNAs and transcriptome data to infer the miRNA–target mRNA relationship changes under dehydration. Strikingly, the authors found 68.4% loss of inverse correlation of miRNA–target mRNA pairs (851/1,245) under dehydration. To understand how high levels of AGO1 correlate to high miRNA–mRNA uncoupling, the authors further investigated conformational changes of AGO1 that might compromise RISC functionality. They hypothesized that AGO1 undergoes condensation and accumulation via its N-terminal prion-like domain (PrLD), an intrinsically low-complexity region rich in aromatic residues that facilitate the formation of condensates through phase separation (Maristany et al. 2024).

To investigate if the PrLD is essential for AGO1 condensation, the authors used the PrLD-containing fragment of AGO1, the PrLD-deleted fragment of AGO1, and full-length AGO1 proteins to see if they undergo LLPS in vitro in the presence of polyethylene glycol (PEG). The PrLD-containing fragment of AGO1 and full-length AGO1 undergo phase separation to form droplets, whereas the PrLD-deleted fragment of AGO1 cannot form droplets. However, the authors reported atypical LLPS behavior in AGO1 droplets, which, under high protein and PEG concentrations, show minimal fusion and size expansion. This implies the possibility that, upon excessive exposure to a dehydrating environment, AGO1 droplets or condensates may transition from a liquid state to a semi-gel-like state. Given that previous studies have reported elevated calcium levels under drought stress (Huang et al. 2018; Li et al. 2022), the authors tested for the formation of droplets of purified AGO1 protein with CaCl_2_ in vitro. Interestingly, Ca^2+^ alone is sufficient to induce the formation of AGO1 droplets, and PEG treatment further enhances the AGO1 droplet formation. Consistently, AGO1 levels are notably increased in *acd* mutants that exhibit highly upregulated cytoplasmic Ca^2+^ levels (Fig. 1A). The authors proposed a model whereby under dehydration stress, Ca^2+^ surge propels AGO1 to form condensates, which prevents the deployment of efficient RISC by uncoupling miRNA–mRNA interaction required for gene silencing (Fig. 1B).

**Figure 1:**
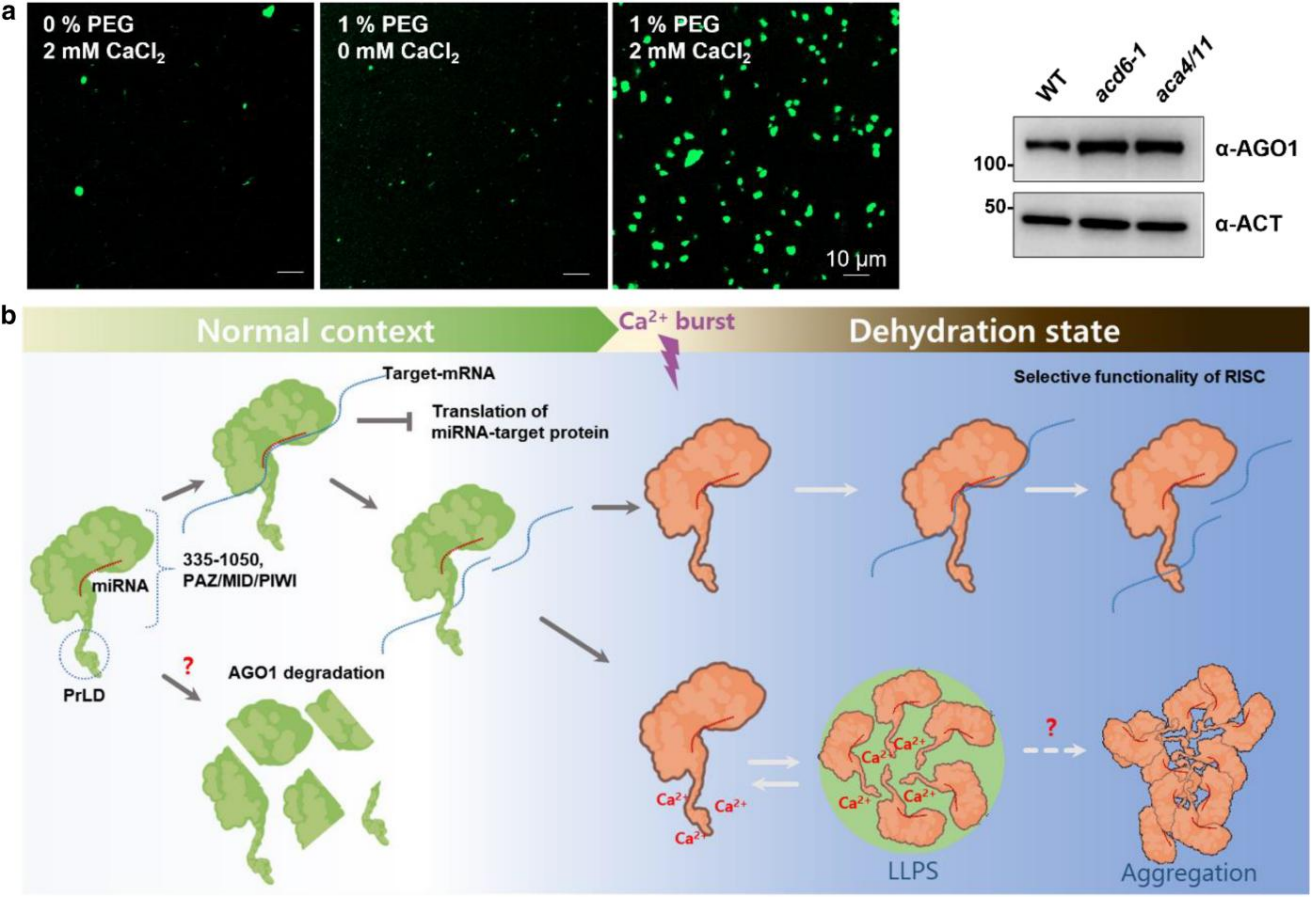
AGO1 forms condensates during dehydration, uncoupling miRNA–mRNA interactions. a) In vitro droplet formation of AGO1 PrLD-containing fragment (1 to 175 aa) with varying concentrations of PEG and CaCl_2_. Formation of condensates of PrLD-containing fragments of AGO1 is plausible with just CaCl_2_ and low percentage of PEG (1%). b) Graphical abstract of the mechanistic model of AGO1 condensate formation under calcium ion surge during dehydration, uncoupling miRNA–mRNA interactions. Figures adapted from Fig. 6A and graphical abstract of Jung et al. (2026).

In summary, Jung et al. (2026) first elucidated the mechanism linking the formation of AGO1 condensates to gene silencing. Their study is also pivotal in elucidating plants' coping mechanisms and stress-mitigation strategies under suboptimal conditions, such as dehydration, rather than just extreme conditions like drought stress, before stress causes detrimental effects on plant development and growth.

## Recent related articles in *The Plant Cell:*

- Li et al. (2024) report that *ALTERED MERISTEM PROGRAM1 (AMP1)* and its putative paralog *AMP*-*LIKE AMP1 (LAMP1)* can impair RNA silencing by repressing the biogenesis of a subset of inverted repeat (IR)-derived siRNAs in Arabidopsis by regulating AGO1 protein levels.
- A review by Genschik et al. (2024) discusses current understanding of the RNA silencing machinery in plant development and microbial hijacking of endogenous proteases, including the discussion on the role of AGO1-RISC complex.
- Legen et al. (2024) studied the PrLD in chloroplast-localized RNA-binding protein CP29A that undergoes temperature-dependent phase separation, which affects RNA splicing and translation in cold-treated tissues of Arabidopsis.

## Data Availability

No new data were generated or analysed in support of this article.
